# A high–throughput digital script for multiplexed immunofluorescent analysis and quantification of sarcolemmal and sarcomeric proteins in muscular dystrophies

**DOI:** 10.1186/s40478-020-00918-5

**Published:** 2020-04-17

**Authors:** Dominic Scaglioni, Matthew Ellis, Francesco Catapano, Silvia Torelli, Darren Chambers, Lucy Feng, Caroline Sewry, Jennifer Morgan, Francesco Muntoni, Rahul Phadke

**Affiliations:** 1 Dubowitz Neuromuscular Centre, UCL Great Ormond Street Institute of Child Health, London, UK; 2 NIHR Great Ormond Street Hospital Biomedical Research Centre, UCL Great Ormond Street Institute of Child Health & Great Ormond Street Hospital for Children NHS Foundation Trust, London, UK; 3 Department of Neurodegenerative Diseases, UCL Queen Square Institute of Neurology, London, UK; 4 School of Cancer Sciences, University of Southampton, Southampton, UK; 5 Dubowitz Neuromuscular Centre, UCL Queen Square Institute of Neurology & Great Ormond Street Hospital for Children NHS Foundation Trust, London, UK; 6 Division of Neuropathology, National Hospital for Neurology and Neurosurgery, UCLH NHS Foundation Trust, London, UK; 7 Dubowitz Neuromuscular Centre, Division of Neuropathology, UCL Queen Square Institute of Neurology, Queen Square, London, WC1N 3BG UK

**Keywords:** Dystrophin, Muscular dystrophy, Quantification, Digital pathology, Immunofluorescence, Genetic therapies

## Abstract

The primary molecular endpoint for many Duchenne muscular dystrophy (DMD) clinical trials is the induction, or increase in production, of dystrophin protein in striated muscle. For accurate endpoint analysis, it is essential to have reliable, robust and objective quantification methodologies capable of detecting subtle changes in dystrophin expression. In this work, we present further development and optimisation of an automated, digital, high-throughput script for quantitative analysis of multiplexed immunofluorescent (IF) whole slide images (WSI) of dystrophin, dystrophin associated proteins (DAPs) and regenerating myofibres (fetal/developmental myosin-positive) in transverse sections of DMD, Becker muscular dystrophy (BMD) and control skeletal muscle biopsies. The script enables extensive automated assessment of myofibre morphometrics, protein quantification by fluorescence intensity and sarcolemmal circumference coverage, colocalisation data for dystrophin and DAPs and regeneration at the single myofibre and whole section level. Analysis revealed significant variation in dystrophin intensity, percentage coverage and amounts of DAPs between differing DMD and BMD samples. Accurate identification of dystrophin via a novel background subtraction method allowed differential assessment of DAP fluorescence intensity within dystrophin positive compared to dystrophin negative sarcolemma regions. This enabled surrogate quantification of molecular functionality of dystrophin in the assembly of the DAP complex. Overall, the digital script is capable of multiparametric and unbiased analysis of markers of myofibre regeneration and dystrophin in relation to key DAPs and enabled better characterisation of the heterogeneity in dystrophin expression patterns seen in BMD and DMD alongside the surrogate assessment of molecular functionality of dystrophin. Both these aspects will be of significant relevance to ongoing and future DMD and other muscular dystrophies clinical trials to help benchmark therapeutic efficacy.

## Introduction

There is a growing need for reliable, replicable, automated, high-throughput, digital image analysis techniques that can be utilised for the pathological assessment of diagnostic and clinical trial samples. Improvement in accuracy and reliability of digital analysis platforms presents an alternative to manual, labour intensive and potentially subjective histological assessments, or semi-quantitative expert assessment. When coupled with high-throughput slide scanning to generate whole slide images (WSI), in both brightfield and fluorescent paradigms, digital image analysis facilitates the assessment of entire tissue landscapes, alleviating potential bias from manual selection of discreet regions of interest for analysis, and enabling up-scaled assessment of fibres in a biopsy from a few hundred to many thousands. Subsequent image analysis using modern tissue phenomics software can unlock unparalleled multiparametric data analytics for biological tissues [7, 11, 23, 37]. Alongside diagnostics, there is also a growing interest in the use of automated digital scripts for the analysis of pathological end-points and/or outcome measures in clinical trial samples [35].

During the last decade, there has been a rapid expansion in the number of Duchenne muscular dystrophy (DMD) clinical trials [49]. DMD is a recessive X-linked genetic disorder affecting 1 in 5000 males, caused by mutations in the *DMD* gene that preclude the production of the protein dystrophin [10, 28]. Dystrophin is essential for stabilisation of muscle fibres during contraction by linking the extracellular matrix and myofibre cytoskeleton. This is achieved via interaction of dystrophin with the subsarcolemmal actin network, and many other proteins at the sarcolemma, such as α-sarcoglycan, β-dystroglycan and nNOS, that together form the dystrophin associated protein complex (DAPC) [19]**.** Lack of dystrophin results in severe contraction induced muscle damage, causing continuous cycles of muscle degeneration and regeneration, ultimately leading to depletion of muscle mass, fibrofatty replacement and loss of muscle function in affected patients [21]. Regenerating myofibres re-express developmental and fetal myosin heavy chains – isoforms that are normally highly expressed in embryonic and fetal skeletal muscles [42].

The primary molecular endpoint for many DMD clinical trials and the proof of concept for therapeutic approaches is the induction or increase in the production of dystrophin [30]. For example, exon skipping therapies aim to modulate the pre-mRNA splicing of the *DMD* transcript using antisense oligonucleotides to restore the reading frame of the gene, leading to the production of shortened dystrophin protein [39]**.** Other therapeutic approaches currently being assessed include stop codon read-through agents [47], myoblast transplantation (NCT02196467) and more recently gene replacement therapy using adeno associated viruses (AAVs) [16, 18]. Irrespective of the individual therapeutic approach, immunohistochemical evaluation and quantification of sarcolemmal dystrophin is a key pathological outcome measure. To evaluate the molecular efficacy and success of DMD clinical trials, robust, reliable and objective methodology for dystrophin quantification must be utilised [3, 6, 35]. Furthermore, there is a growing interest to not only localise and quantify the level of restored dystrophin in myofibres of post-treatment biopsies, but also to correlate its levels to key DAP interactions, to demonstrate surrogate molecular functionality [14, 29, 30]. The field has gradually evolved from semi quantitative dystrophin quantitation [5, 44], requiring a significant manual interface, to more recent attempts at fully automated whole section quantitation [4, 38]. While whole section digital analysis allows for greater precision and coverage in quantification, it is highly susceptible to the myriad of tissue processing artefacts, as well as the inherent arbitrariness in the setting of exposure times for image capture and strategies employed to minimise non-specific background signal. In addition, many scanners may not be calibrated in the same way as traditional fluorescent microscopes to account for day to day variability. It is therefore essential for automated image analysis systems and digital scripts to be suitably optimised and validated for use in a clinical trial environment [3]. Furthermore, images must be of a consistent quality having been immunostained or histochemically stained and acquired via highly reproducible methods [1, 3, 4, 35, 38].

Through a previous study, the foundation for a high-throughput, operator-independent digital method was laid; this focussed on the sarcolemmal intensity of dystrophin through analysis of multiplexed laminin or spectrin, providing the membrane mask, with dystrophin [38]. The technique was capable of analysing virtually all intact myofibres in a transverse section, giving novel insight into the significant variability of dystrophin expression between muscle fibres in the same section not only in BMD and DMD samples but also in healthy controls. A statistically significant difference in mean dystrophin intensity was observed between two different dystrophin antibodies (ab15277 and MANDYS106) highlighting the critical influence of choice of antibody for immunolabeling and acquisition methods in determining the outcome of quantitative analysis. However, in this study strategies for effectively addressing correction of background signal, optimisation of exposure times and acquisition parameters, and surrogate demonstration of the molecular functionality of induced dystrophin were not systematically addressed.

To address some of these needs, a new series of image analysis methods implemented in Definiens Developer XD (Munich) have been developed, applicable to digital scans of entire sections of skeletal muscle and tailored specifically to the assessment and study of DMD and BMD biopsies. Data generated from this new method allows not only quantification of dystrophin at the sarcolemma but provides vital information of colocalisation and quantification of key DAPs in relation to sarcolemmal dystrophin. Furthermore, the acquisition procedure has been refined and a novel method for background subtraction has been implemented in an attempt to accurately identify all true dystrophin signal present at the sarcolemma. In addition, automated assessment of the levels of myofibre regeneration as assessed by the number of fibres expressing fetal and/or developmental myosin is presented [24, 40–42], as it is expected that a robust increased production of sarcolemmal proteins like dystrophin (or utrophin) would be associated with decrease in muscle damage [22, 24].

## Methods

### Muscle sectioning

Serial unfixed frozen sections (7 μm-thick) were cut from 3 control, BMD and DMD frozen muscle biopsies with a Leica CM 1850 UV cryostat (Leica Biosystems, Germany). Slides were air dried for 45 min before staining and a Super pap pen (Daido Sangyo LTD, Japan) was used to create hydrophobic barriers around the tissue.

### Immunostaining

Samples were incubated with 200-300 μl of primary (1 h-RT) and 200-300 μl of secondary (30 min-RT) cocktail of antibodies diluted in PBS (Fisher Scientific, UK). Volumes varied based on the size of the tissue sections to ensure they were completely covered. Details of the antibody combinations and specifications used are listed in Table 1. A cocktail of developmental and fetal/neonatal myosin antibodies (designated f/d myosin cocktail) was employed to enable recognition of fibres in all stages of regeneration and/or aberrant re-expression of these immature myosin heavy chain isoforms in dystrophic myofibres. Sections were washed for 3 × 3 min with PBS, before and after the incubation with secondary antibodies. Stained sections were mounted with Hydromount (National Diagnostics, UK) and cover slipped with 22 × 26 mm cover glasses (VWR, Belgium). Slides were stored at 4 °C until acquisition. For each sample, 2 serial sections were stained in duplicate for each experiment.

**Table 1:**
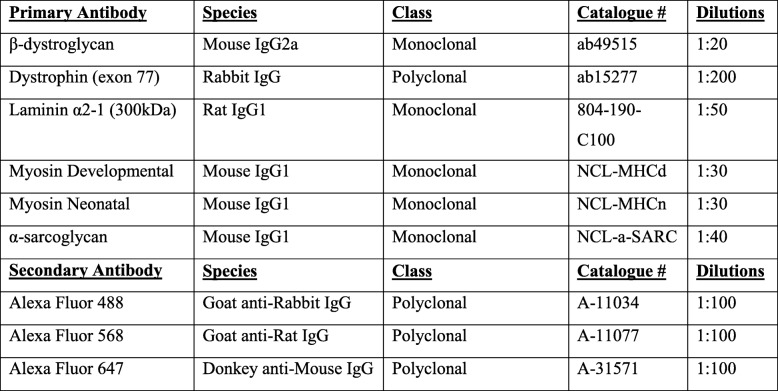
Antibodies

All antibodies used with information on species, isotype, class, catalogue number and working dilution. Figure shows antibody combinations used for **(a)** α-sarcoglycan **(b)** β-dystroglycan and **(c)** f/d myosin triple stains

### Acquisition

Slides were scanned within 24 h of immunostaining on a ZEISS Axio Scan.Z1 Slide Scanner (Carl Zeiss Microscopy GmbH, Germany). Laminin α2 staining in the 568 channel was used as a reference protein for coarse and fine focus map generation using the 10x and 20x objectives respectively. After creation of the focus map, 3 channel (488, 568 and 647) WSIs were captured using the 20x objective with offline stitching. The scanner used for this study is calibrated annually with its LEDs measured to an internal reference standard to ensure stable and constant output power over the entire lifetime of each LED. Additionally, as long as only one LED is turned on, the photodiode acts as a closed-loop power stabilization in the μs range. It is capable of controlling the power of the active LED even during the exposure time of an image. This guarantees a stable light output even if the LED is used in short millisecond ranging exposures.

### Statistical analysis

One-way ANOVA test with Tukey's multiple comparison correction was used to compare means of each sample with the means of every sample for each experimental condition. GraphPad Prism 7.0 software was used for statistical analysis and graph design. Data are presented as mean ± standard deviation.

### Digital image analysis script methodology

The method described was developed for the analysis of WSI of immunofluorescent (IF) stained transversely cut muscle sections, composed of a mask stain (laminin α2) identifying the myofibre boundary (basal lamina), dystrophin stain, and either one of two additional relevant sarcolemmal markers (α-sarcoglycan or β-dystroglycan), or a sarcoplasmic markers (f/d myosin). The image analysis processing is comprised of three distinct stages: identification of muscle tissue and exclusion of artefacts; identification of muscle fibres within the tissue; and characterisation of morphological features and staining profiles of individual muscle fibres. The analysis has been implemented using Definiens Developer XD (Munich), version 2.7.0. A workflow of the methodology can be found in supplementary file 1. A summary of the salient features of the analysis is outlined below.

#### Digital characterisation of Myofibre morphology and immunohistochemistry

For each identified myofibre, a series of morphological descriptors and staining characteristics were calculated and recorded. The morphological descriptors include fibre area, sarcoplasm area, sarcolemma area, fibre width, sarcoplasm width, mean and maximum sarcolemma thickness. The thresholding for sarcolemma thickness was used to optimise the recognition of true myofibre-rich regions by the mask, and not used as a variable endpoint in measuring dystrophin intensity. The staining characteristics included both direct measurements of staining intensity within the sarcolemma and sarcoplasm for all image channels, and empirically enhanced characteristics. The reporting of stain properties is adapted to the two stain combinations under investigation: mask with primary and secondary sarcolemma stain (dystrophin + α-sarcoglycan/β-dystroglycan); and mask with a primary sarcolemma and secondary sarcoplasmic stain (dystrophin + f/d myosin).

#### Background subtraction and identification of positive sarcolemmal staining

For all sarcolemma stains a background subtraction method was developed to identify only significant levels of staining as positive. A number of background removal methods were investigated, such as global thresholds based on the intensity of staining within non-mask regions or within sarcoplasmic regions, but this was insufficient due to the heterogeneity of staining often observed [38] (see generation of *mask-mod* layer, Supp. file 1).

The selected method was used to threshold for each fibre individually, by using a threshold of significant sarcolemmal staining compared to the cytoplasm (where no functionally relevant protein is expected to be present). Fluorescence intensity was reported as raw values for the sarcolemma (without subtracting the cytoplasmic background). In thresholding for sarcolemmal positivity, it was assumed that the cytoplasmic and sarcolemma region background signal values are equal. Direct comparisons between cytoplasmic background and sarcolemmal background would not be possible due to masking of the sarcolemmal background by true dystrophin signal. By utilising this method, we do not need to set arbitrary intensity thresholds for positivity and are able to adapt dynamically to variations across the section in background fluorescence and non-specific signal.

The Circumference Positivity (the sum of (border length-2)/2 for each positive object in a fibre, as a proportion of the circumference of sarcolemma) was used to provide coverage measurements as it has been shown to provide both quantitatively and qualitatively robust measurement of positive sarcolemmal protein coverage, providing a high degree of consistency between similarly scored fibres and with pathologist classification.

Each fibre was then classified into one of 4 classes according to the Circumference Positivity: 0–25%, 25–50, 50–75% and 75–100% coverage. Fibres with 0–25% positivity were classified as protein negative whilst fibres with > 25% coverage as protein positive.

The 25% or more sarcolemmal coverage cut off used for defining binary ‘protein positive/negative’ fibres was arrived at after a pathologist’s review of classification results set at different percentage thresholds. Images with a threshold < 25% were observed to produce greater false-positive characterisation, including classification of ‘non-muscle’ elements in the section, in addition to trace dystrophin at the sarcolemma. We therefore opted for a conservative threshold of 25%, similar to that of other studies, as this minimises the inclusion of ‘false-positive’ sarcolemmal coverage for dystrophin and DAPs [4]**.** Similar assessment was performed for different thresholds of identification of positive sarcolemmal stain. The final formula provided the greatest level of concordance with a pathologist’s manual assessment of the raw fluorescent image against the generated classification map for protein positive and protein negative fibres (Fig. S5). The intensity of the primary (dystrophin) and secondary (α-sarcoglycan/β-dystroglycan) sarcolemmal stains were calculated in the dystrophin positive and dystrophin negative objects contained within the sarcolemma for each fibre. This provided measurement of the secondary sarcolemmal stain within the sarcolemma region classified as positive and negative for the primary sarcolemma stain, which provides a direct measurement of colocalisation.

For classification of f/d myosin positive fibres, an average sarcoplasm intensity threshold was applied. This threshold was derived from staining of control samples in which f/d myosin is not present thus acting as a negative baseline. Myofibres with average sarcoplasmic f/d myosin fluorescence intensity greater than the threshold were subsequently classified as positive.

## Method development and optimisation

To facilitate the use of the dystrophin and DAP quantification modules of this script, systematic optimisation steps were performed to determine the most favourable conditions for myofibre immunostaining, image acquisition and final analysis. These included: determining ideal combinations of fluorophores and filters for triplex immunoassays, with no antibody cross-reaction or fluorescent bleed through and discerning optimal acquisition exposures, allowing for the most accurate final analysis. The aim of this optimisation is to achieve greater levels of reproducibility and reliability by maintaining consistent exposure times across independent experiments, rather than altering them on a batch to batch basis as was done previously [38].

Once optimal conditions for acquisition and their stability were established, quality checks were performed on the script itself, overseen by an experienced muscle pathologist, to ensure good concordance between manual assessment and fully automated analysis of the digital images. Once established, the optimal acquisition parameters and exposure times were kept constant for the duration of the study.

The first step in method development was to find optimal exposure settings that enabled visualisation of trace dystrophin and DAPs in DMD samples but did not saturate the fluorescence signal obtained when proteins are in abundance in control samples. The previously used method involved setting variable exposure times for each experiment based on the signal generated from a control sample. However, it is now widely accepted that many myofibres of DMD patients produce trace amounts of dystrophin that correctly localises to the sarcolemma [1, 6, 9]. Using shorter exposure times, based on generating optimal signal of a control sample, often results in the inability to detect this trace dystrophin and also the underestimation of subtle, restored dystrophin following therapeutic intervention. The following strategy was implemented to minimise variability in exposure settings and strike a balance between signal saturation and under-reporting.

All samples were immunostained for 3 proteins of interest; laminin α2 (acting as a mask for myofibre identification), dystrophin and a tertiary protein marker of interest (α-sarcoglycan, β-dystroglycan or f/d myosin). Four regions were randomly selected from CTRL, BMD and DMD samples and ‘auto exposure’ values were generated by the Zeiss AxioScan fluorescent slide scanner for dystrophin and each tertiary protein marker. The auto-exposure values obtained for each protein were then averaged in controls, BMD and DMD samples, along with a global average from all samples.

Separate images were then captured using the 4 different exposure times (the average of CTRLs, average of BMDs, average of DMDs and the global average) and subsequently assessed visually by the pathologist to gauge the final ‘optimal’ exposure setting that would allow detection of a range of dystrophin signal from trace to bright ‘revertant’ fibres (DMD myofibres strongly positive for dystrophin due to natural restoration of the reading frame due to exon skipping during transcription [45]) with minimum background, recapitulating the staining pattern ascertained from diagnostic experience. This was determined to be the ‘global average’ as it allowed good visual assessment of trace dystrophin present throughout the DMD samples but did not saturate the fluorescence signal generated in the controls when analysed via the script. The same method was applied for α-sarcoglycan and β-dystroglycan tertiary protein markers. The global average was again determined to be most optimal for protein visualisation in the BMD and DMD samples without saturating the signal in the CTRLS. For f/d myosins, the average signal obtained from the BMD and DMD samples was used, as the CTRL samples do not express the protein.

## Results

### Consistency and reproducibility

**Fig. 1:**
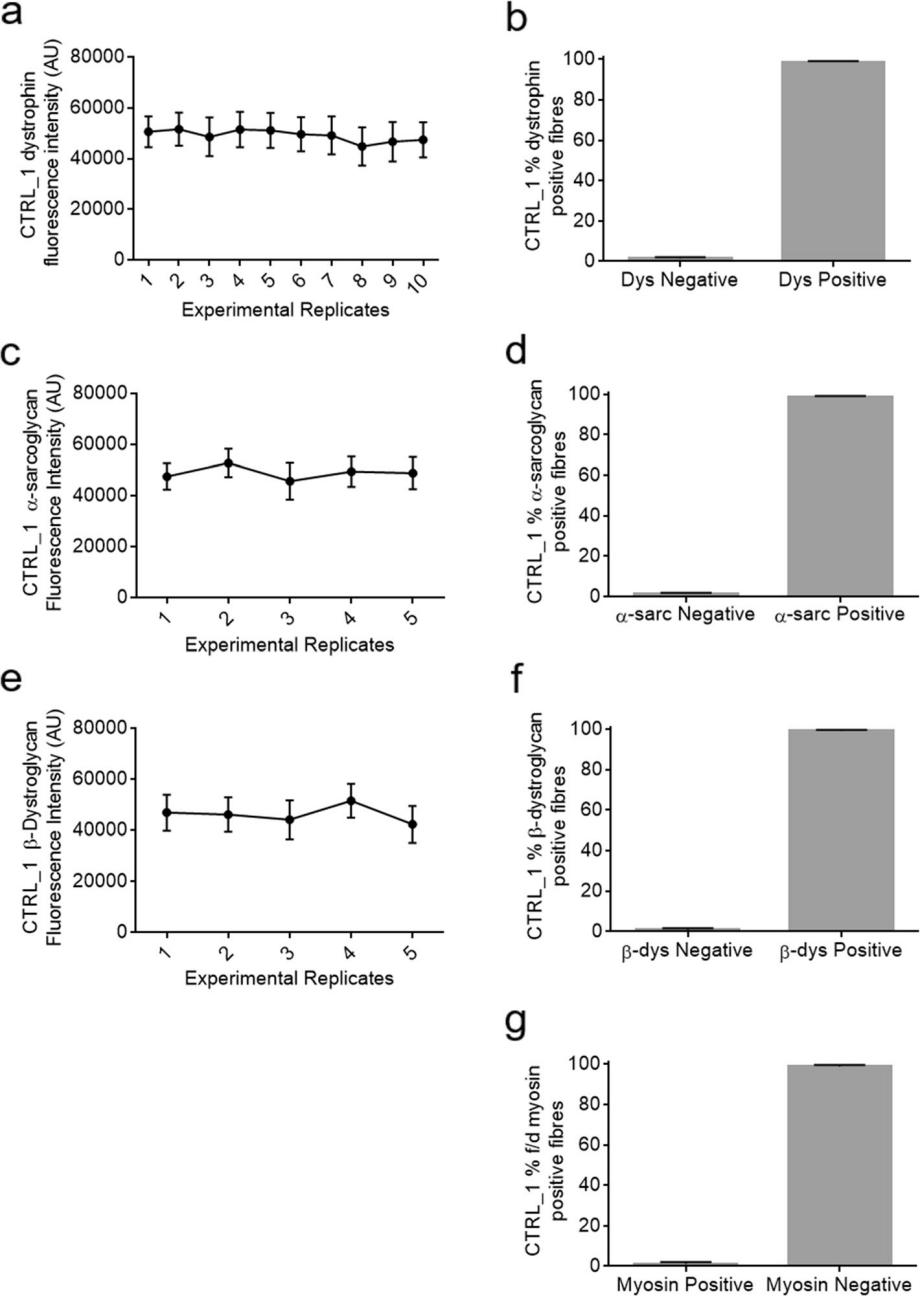
Serial sections from CTRL_1 were immunostained for dystrophin (**a**-**b**), a-sarcoglycan (**c**-**d**) and B-dystroglycan (**e**-**f**) separately on different occasions over a 5-week period. Each slide was immunostained according to the same protocol and acquired at the AxioScan with the same exposure times and configurations. Images were processed with the script to assess natural variability in immunostaining, acquisition and analysis. Fluorescence intensity and % positive myofibres were calculated for all images. Serial sections from CTRL_1 were also stained for f/d myosin to determine if false positive myofibres were being incorrectly identified during the analysis (**g**)

To first assess consistency and reproducibility of both the finalised laboratory method and finalised digital script analysis, serial sections from a CTRL sample (CTRL_1) were immunostained and analysed on 10 separate days across a 5-week period. 5 sections were stained for dystrophin, laminin α2 and α-sarcoglycan while 5 sections were stained for dystrophin, laminin α2 and β-dystroglycan. Across the 10 replicates the average mean dystrophin intensity was 49,119 AU (arbitrary units) with a maximum and minimum average of 51,662 and 46,669 respectively. This represents a good degree of reproducibility with only 10% variability in fluorescence intensity across the entire period (Fig. 1a**).** When assessed for % positivity of dystrophin, all 10 replicates gave an average of 99% positivity with minimum and maximum values of 98.5 and 100% positive **(**Fig. 1b).

Similar results were observed for replicate analysis of α-sarcoglycan and β-dystroglycan **(**Fig. 1c-f**)**. Minimal variation in mean fluorescent intensity was seen across the 5 replicates for each protein with each sample consistently returning 98–100% protein positivity for analysed fibres, in accordance with manual observation. Maximum and minimum intensity values were 45,603 AU and 52,730 for α-sarcoglycan analysis and 44,074 and 51,533 for β-dystroglycan. Variation in α-sarcoglycan intensity was 13.5% across the 5 experiments while variation in β-dystroglycan was 18%.

For f/d myosin analysis, 5 replicates from the same control sample were immunostained for laminin α2 and a cocktail of f/d myosin on 5 separate days. Each experiment returned a minimum of 0% positive and a maximum of 2% positive f/d myosin positive fibres. **(**Fig. 1g**).** This demonstrated the script was not aberrantly detecting an abundance of false positive regenerating myofibres that are not present in healthy samples and that a reliable threshold for positivity had been set.

### Dystrophin analysis

#### Variable patterns of dystrophin fluorescence intensity between different patients and disease categories

Having determined the optimal technical methods for immunostaining, acquisition and quantification, we then performed a small proof of concept study using images generated from 3 CTRL, 3 BMD and 3 DMD samples. The levels of dystrophin, DAPC stain and f/d-myosins were quantified and demographics for all samples are listed in Table 2.

**Table 2:** Sample demographics

Sample	Age at Biopsy	Mutation	Diagnosis	Age at onset of symptoms (years)	Motor and cognitive function
CTRL_1	9y6m				
CTRL_2	14y0m				
CTRL_3	7y10m				
BMD_1	7y7m	Mutation in intron 14 (C.1705-18 T > G) resulting in abberant splicing of exon 15 (predicted in frame)	BMD CK 1700	Age 8 with tiredness on running	Autistic spectrum disorder Aged 15 can walk for 30 min, but more slowly compare to his peers. He continues hower to remain ver active, for example at school plays football, badminton and basketball
BMD_2	3y2m	Deletion exons 45–47	BMD		Walks with a waddle. Can just about run but is unable to hop. Gets up with a modified Gower’s manoeuvre.
BMD_3	9y0m	Deletion exons 3–7	BMD CK 4117	Age 9 with a history of muscle weakness	Problems running and difficulty getting up off the floor. Unable to hop and has difficulty climbing stairs.
DMD_1	4y8m	Duplication of exons 5–7	DMD, diagnosed at 2.5 for incidental finding of high CK (28,000)	3.5 years	Steroids declined. Lost of ambulation age 8 years 10 months
DMD_2	6y10m	Deletion of exons 6–44 (predicted in-frame)	DMD CK 25500	4 years, with peak of activity aged 6 and deterioration from age 7	Lost ambulation aged 10; special education needs. On steroids
DMD_3	3y3m	Hemizygous mutation, c.4517_4518delTG (p.Val1506fs) in exon 32	DMD CK 15189	3 years	Behaviorual difficulties. Age 10 walks slowly for up to 30 min.

Demographics for all control and patient samples used in this study

**Fig. 2:**
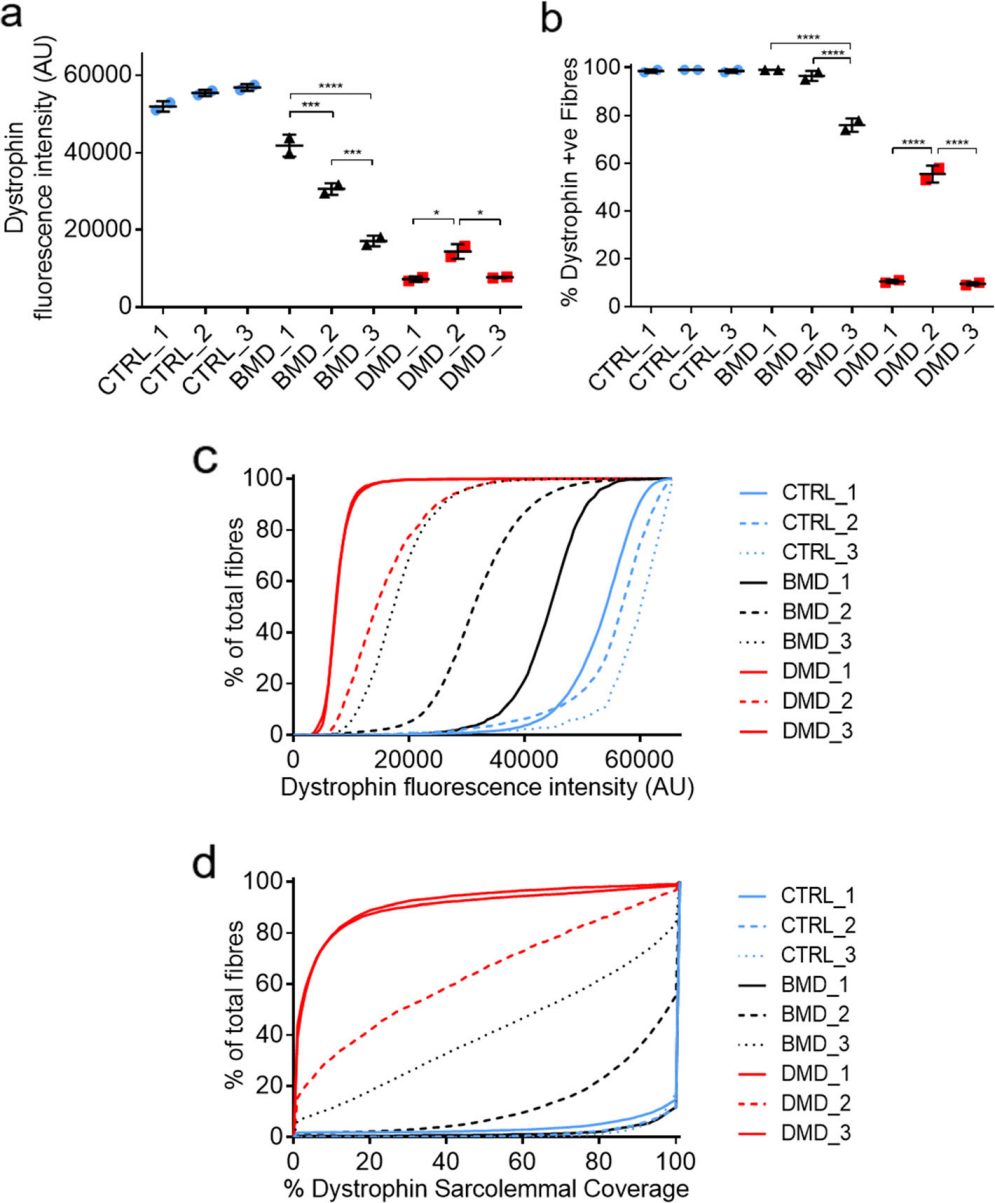
**a** Mean sarcolemmal dystrophin intensity based on sarcolemmal fluorescence analysis of dystrophin immunostaining**. b** % myofibres positive for dystrophin immunostaining. A positive fibre is classified as having > 25% sarcolemmal circumference coverage for dystrophin immunostaining. **c** Cumulative frequency graph for sarcolemmal dystrophin fluorescence intensity and **d** cumulative frequency graph for % of positive sarcolemmal dystrophin circumference coverage in all samples. All samples were immunostained and acquired at the same time under the same conditions. *N* = 2 serial sections for each sample

All CTRL, BMD and DMD samples were immunostained for laminin α2 and dystrophin with each sample containing 2 serial sections to act as technical replicates. Mean dystrophin fluorescence intensity, % of dystrophin positive fibres and the cumulative frequency of sarcolemmal dystrophin circumference coverage was then assessed. The average fluorescence intensity of the 3 CTRL samples was 54,759 AU, BMDs was 29,829 AU and DMDs was 9686 AU **(**Fig. 2a**).** The percentage variation within each group was 9, 59 and 50% respectively for CTRL, BMD and DMD samples, with no statistically significant variation between CTRL samples. However, significant variation was observed between all BMD samples, and also observed between DMD_1/DMD_3 and DMD_2. This variation can also be observed when intensity is plotted as a cumulative frequency against percentage of total fibres for each sample **(**Fig. 2c**)**.

#### Variable patterns of dystrophin positivity between different patients and disease categories

For percentage positivity (> 25% dystrophin immunostaining at the sarcolemma), CTRLs showed an average of 99% dystrophin positive myofibres, BMDs 91% and DMDs 25%. However, more substantial variability between individual patients was apparent, with BMD_1 showing 99% positivity compared to 76% for BMD_3. This variability was also apparent for the DMD samples, where DMD_1 and DMD_3 showed similar 11 and 10% positivity, respectively, while DMD_2 showed a substantially higher 56% dystrophin positive fibres **(**Fig. 2b**)**. The percentage variation within each sample group (CTRLs, BMDs and DMDs) was 0.5, 23.2 and 83.8% respectively. Significant differences in percentage positivity within each disease group were observed between BMD_1/BMD_2 and BMD_3 and DMD_1/DMD_3 and DMD_2. No significant differences were observed between CTRL samples.

**Fig. 3:**
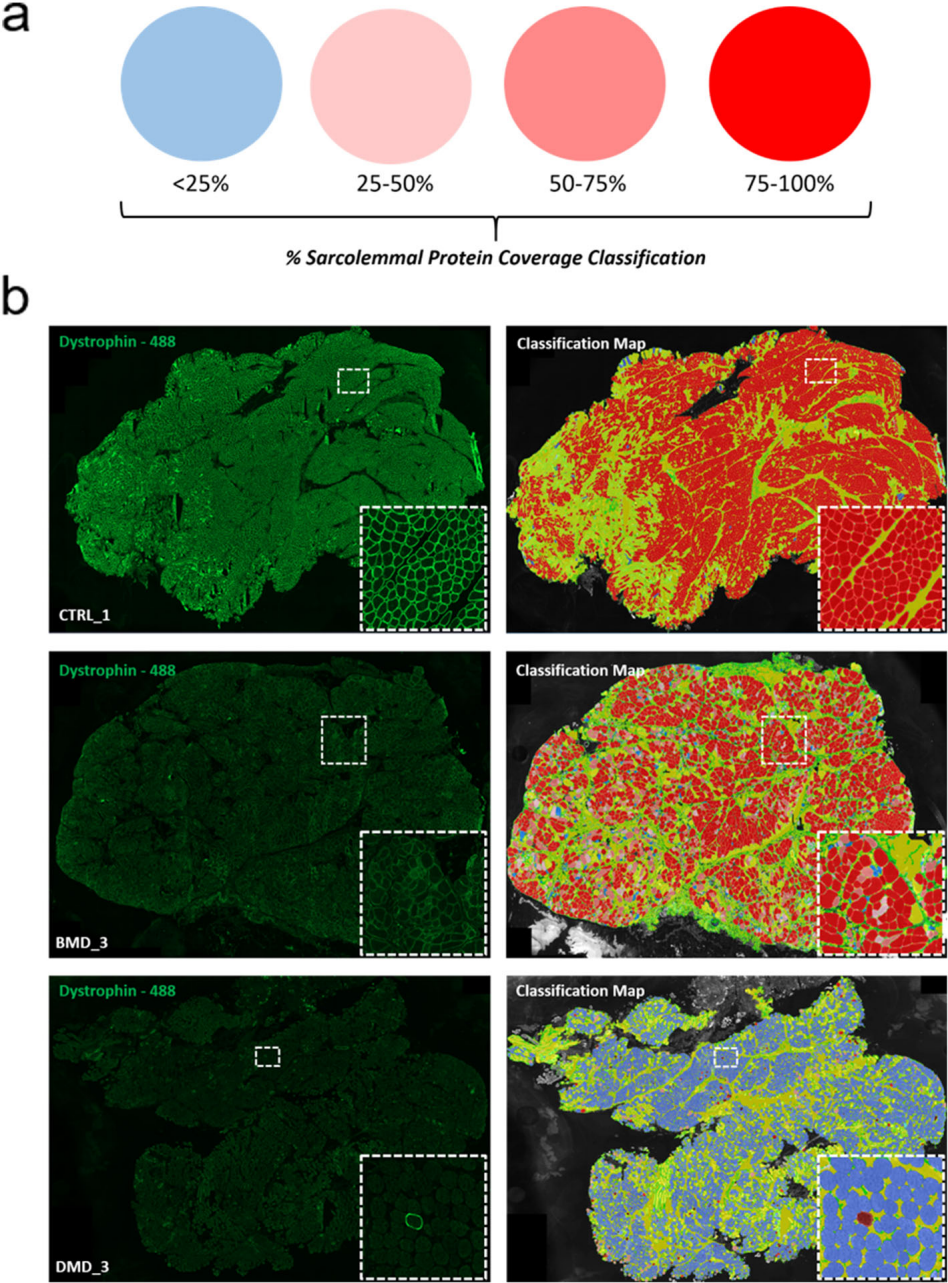
**a** Myofibre classification key for coverage of sarcolemmal protein markers (Dystrophin, α-sarcoglycan or β-dystroglycan). Sarcolemmal circumference coverage is used to place fibres in 4 categories. Protein negative (< 25% coverage) or varying degrees of protein positive (25–50%, 50–75% or 75–100% sarcolemmal circumference coverage). **b** Image panel highlighting dystrophin immunofluorescence staining (green 488) of CTRL_1, BMD_3 and DMD_3 and their corresponding dystrophin classification maps. Yellow regions show connective tissue or myofibres have not been recognised/detected during the fibre recognition phase

This variation is observed better when the sarcolemmal circumference coverage for dystrophin positivity is plotted as a cumulative frequency graph **(**Fig. 2d**)**. Here, variation in levels of percentage positivity among the BMD and DMD samples can more clearly be observed. DMD_1 and DMD_3 follow very similar profiles while DMD_2 shows a similar pattern of positivity to BMD_3. All 3 CTRLs show uniformity in their percentage coverage in comparison to the BMD and DMD samples. Variation can also be observed between BMD_1 and BMD_2 which was not as clearly apparent when simply analysing % positive fibres (> 25% coverage of dystrophin). In addition, for each sample, classification maps of dystrophin positivity are also generated to highlight fibre variability in percentage coverage across the entire section and provide a visual map of the analysed data. Sarcolemmal circumference coverage was used to place fibres in 4 categories. Protein negative (< 25% coverage) or varying degrees of protein positive (25–50%, 50–75% or 75–100% sarcolemmal circumference coverage). Fig. 3 shows examples of the dystrophin immunostaining and corresponding dystrophin classification maps for CTRL_1, BMD_3 and DMD_3. Uniform 75–100% myofibre dystrophin circumference coverage can be seen in CTRL_1 while BMD_3 shows greater heterogeneity of coverage. DMD_3 shows predominantly negative coverage, consistent with the lack of dystrophin on most fibres.

### DAPC analysis

Alongside primary analysis of dystrophin, we analysed key proteins within the DAPC that interact with dystrophin. Samples were immunostained for a combination of laminin α2, dystrophin, and either α-sarcoglycan or β-dystroglycan.

**Fig. 4:**
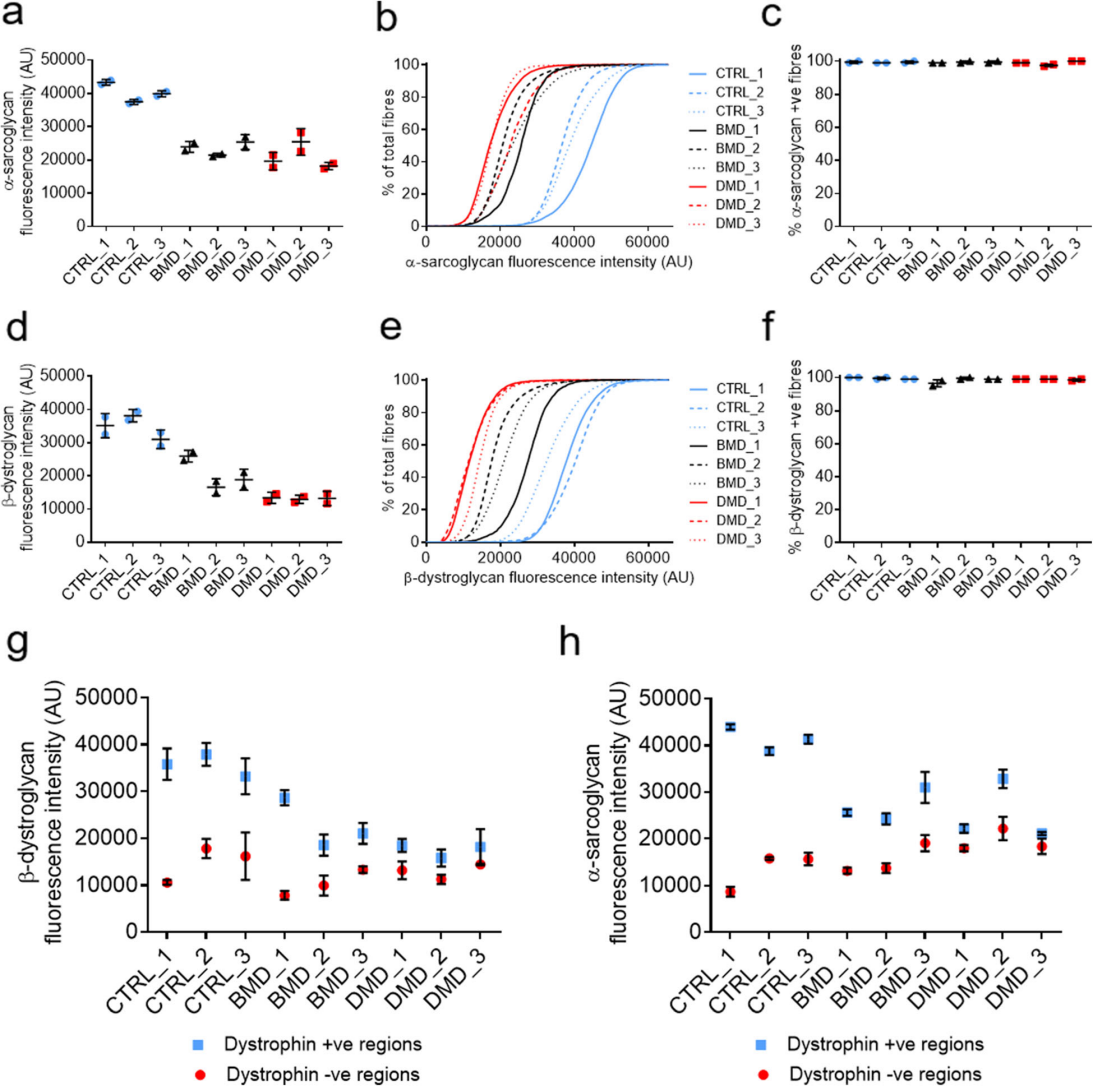
Mean fluorescence intensity of sarcolemmal α-sarcoglycan (**a**) and β-dystroglycan (**d**). Cumulative fluorescence intensity for α-sarcoglycan (**b**) and β-dystroglycan (**e**)**.** Average % myofibres positive for α-sarcoglycan (**c**) and β-dystroglycan (**f**). A positive fibre is classified as having > 25% sarcolemmal protein circumference coverage. β-dystroglycan (**g**) and α-sarcoglycan (**h**) intensity in regions of sarcolemmas that were classified as either positive or negative for dystrophin. All samples were immunostained and acquired at the same time under the same conditions. *N* = 2 serial sections for each sample

Mean and cumulative sarcolemmal fluorescence intensity of α-sarcoglycan/β-dystroglycan was assessed **(**Fig. 4a, b, d, and e**)** alongside the % positivity of myofibres for either protein in each sample **(**Fig. 4c**+f)**. Additionally, fluorescence intensity of these tertiary markers (α-sarcoglycan and β-dystroglycan) was assessed in regions of myofibre sarcolemma that has been classified as either dystrophin positive or dystrophin negative **(**Fig. 4g-h**)**. This paired, ordered processing allows comparative quantification of the level of DAPs in the presence or absence of dystrophin at the sarcolemma.

Average CTRL mean fluorescence intensity for α-sarcoglycan and β-dystroglycan was 40,268 AU and 34,773 AU respectively with 13.5 and 16.4% variation between the 3 CTRL samples. Average BMD intensity was 23,615 AU (α-sarcoglycan) and 20,458 AU (β-dystroglycan) with 15.5 and 36.1% variation between samples while the average DMD values were 21,096 AU (α-sarcoglycan) and 13,153 AU (β-dystroglycan) **(**Fig. 4a**+d)** with 28.6 and 2% variation. However, variation in fluorescence intensity within the CTRL, BMD and DMD groups was not statistically significant. All samples showed 98–100% myofibre positivity for both proteins with no significant differences observed **(**Fig. 4c**+f).**

When assessing colocalisation with dystrophin, all samples showed an increase in fluorescence intensity for both DAPs in regions of the sarcolemma identified as dystrophin positive compared to dystrophin negative regions (Fig. 4g-h**).** DMD_2 showed a greater increase in fluorescence intensity of α-sarcoglycan in dystrophin positive regions than DMD_1 and DMD_3. However, this difference was not observed when assessing levels of β-dystroglycan intensity in dystrophin positive regions.

### Myofibre regeneration

A f/d myosin cocktail was used to identify myofibres undergoing regeneration. The number of f/d myosin positive fibres was then determined to give percentage positivity values for the entire section, giving an overall assessment of the regeneration in each sample.

**Fig. 5:**
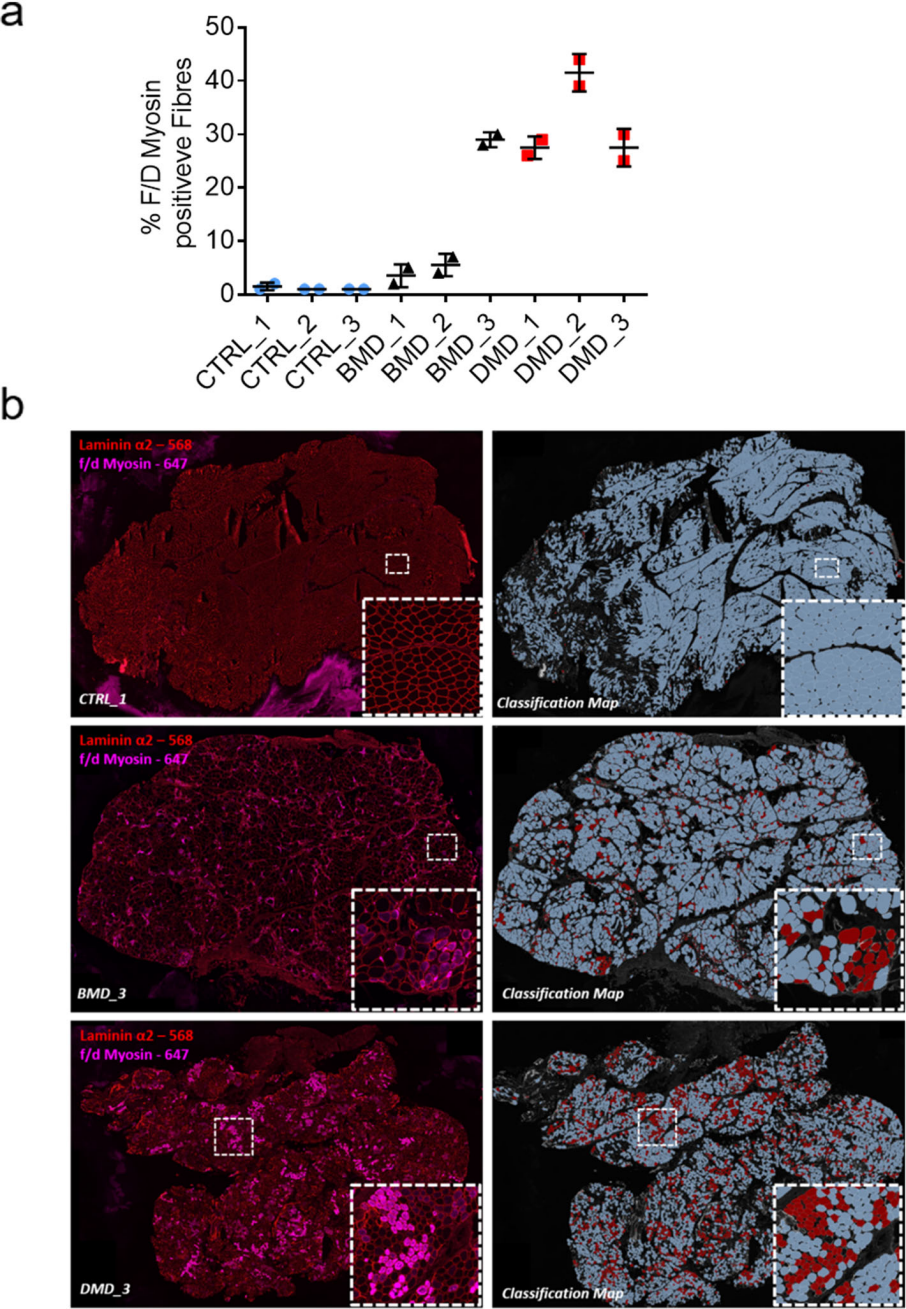
**a** Percentage of fibres in each sample that were classified positive for the presence of fetal and developmental myosin. All samples were immunostained and acquired at the same time under the same conditions. *N* = 2 serial sections for each sample. **b** Image panel highlighting f/d myosin (pink 647) and lamininα2 staining (red 568) of CTRL_1, BMD_3 and DMD_3 and their corresponding f/d myosin classification maps generated via digital analysis. Blue fibres in the classification map are negative for f/d myosin whilst red fibres have been classified as positive

As expected, all 3 controls showed little to no f/d myosin positive fibres with an average value of 1% positivity. BMD_1 and BMD_2 had 4 and 6% positivity respectively whilst BMD_3 presented with 29% of fibres positive for f/d myosin. The 3 DMD samples had a combined average of 32% myofibre positivity for f/d myosin with DMD_2 having the highest value of 42% **(**Fig. 5a**).** Fig. 5b provides representative examples of f/d myosin immunostaining in CTRL_1, BMD_3 and DMD_3 along with their corresponding myosin classification maps generated via digital analysis. Within this classification map, f/d myosin positive fibres are designated in red and negative fibres in blue.

## Discussion

With the rapid advancement in the number of DMD clinical trials, and several drugs recently approved (Exondys 51 [2] and Vyondys 53 [15]) or currently in clinical trials, there is a pressing need for objective and reliable quantification of dystrophin. Dystrophin quantification has been a longstanding challenge within the neuromuscular community [3]. This is primarily a result of its large size (427kDA) [20], the absence of a reliable reference standard [8] and the need to set arbitrary thresholds when analysing fluorescently stained samples to distinguish true signal from background fluorescence [4, 5, 44]. This makes accurate quantification of dystrophin produced as a result of therapeutic intervention challenging via current conventional methods. This challenge is further compounded by the very low and variable expression of induced dystrophin that has been observed in recent trials involving antisense oligonucleotides [13, 14, 17, 25, 26, 46] coupled with the observation that most DMD patient samples also have low level residual dystrophin expression at baseline, highlighting the necessity for a highly sensitive detection method capable of detecting dystrophin signal over a wide dynamic range to resolve even subtle differences between pre- and post-treatment biopsies [3]. The amount and distribution pattern of induced dystrophin needed for functional benefit in humans is not yet known although it is suggested that levels as low as 30% could be sufficient to completely avoid muscle weakness [33], while lower levels are expected to reduce the rate of muscle damage. Indeed, in dystrophin and utrophin knockout mice models, as little as a 5% increase in dystrophin production has been shown to elicit improved survival, muscle function and histology [20, 36, 43], and in recent clinical trials, lower levels were detected in patients receiving eteplirsen, with associated clinical benefit [13, 14, 27, 48]. As a result, it is vital to detect even small increments in protein levels in clinical trial samples as this may be sufficient to generate functional benefit. The goal of many DMD trials is to emulate a ‘BMD-like’ molecular and clinical phenotype. Observational pathology from diagnostic muscle biopsies reports a highly variable dystrophin expression pattern in BMD samples from patients with different mutations in *DMD*. However, this heterogeneity of protein expression has so far been poorly characterised using formal quantitative techniques. It is essential to understand the varying levels of dystrophin expression in different BMD patients as this will be vital to help benchmark the success of therapeutic interventions. We have shown that in just 3 patients for each disease group, there is considerable heterogeneity with differences observed in dystrophin intensity, percentage coverage and amounts of DAPs. Analysis of a larger cohort of BMD biopsies employing the current method is ongoing, aimed at characterising the heterogeneity of dystrophin expression, mapping it to the individual genotypes, and correlating to clinical outcome. This will be vital for future clinical trials in DMD, allowing for more accurate benchmarking of results against well characterised BMD phenotypes.

Analysis of immunostained muscle fibres for dystrophin is complementary to other techniques such as western blotting and mass spectrometry, providing different insights into the molecular efficacy of therapeutic intervention [1, 12]. This is mainly due to the ability to assess dystrophin localisation at the sarcolemma of myofibres which is not achievable by either western blot or mass spectrometry. Furthermore, immunostaining provides the opportunity for multiplex analysis to correlate dystrophin expression directly with other proteins of interest, such as those present in the DAPC or to levels of f/d myosin expression. Quantification of restored dystrophin via western blot has been challenging, primarily due to the need to detect very subtle changes in expression at the lower end of the detection range [3, 13, 27, 46]**.** Immunostaining may allow visualisation and detection of trace dystrophin with greater sensitivity than is achievable with conventional western blotting techniques [46] [44]. However, capillary western immunoassays are now being assessed for quantification of dystrophin over a larger dynamic range using considerable less starting material than conventional western blotting techniques [8]. Another key advantage of western blots over immunohistochemistry is the ability to determine molecular weight of the induced dystrophin.

Here we have presented a novel technique for dystrophin quantification that addresses the lacunae identified in previous methods, including strategies for minimising variability, correction of non-specific background signal, and accurate classification of low level dystrophin signal [4, 38].

As has become custom in the scientific community, exposure times for dystrophin acquisition are usually calculated based on the generation of an optimal signal on control samples with ‘normal’ dystrophin expression [5, 9, 38]. However, this method tends to generate shorter exposure values that may be insufficient to detect the low-level ‘trace’ dystrophin signal that is present on many myofibres in DMD [6]. Higher exposure settings based on the global average (average of CTRLs, BMDs and DMDs) were chosen for this study that put the fluorescence intensity of CTRL samples in the upper range of quantification (~ 50,000 AU) but did not saturate the signal (> 65,653 AU). When assessed by an experienced pathologist, the digitally unaltered ‘raw images’ captured at this ‘global average’ higher exposure setting, most faithfully recapitulated the dystrophin expression pattern as observed under an epifluorescent microscope used in routine diagnostics. This enables visualisation and quantification of low-level dystrophin that presents with very weak fluorescence intensity (~ 8000 AU) that would fall below the lower threshold of the dynamic range if lower exposure times were used**.** This is apparent in the dystrophin analysis of DMD_2. If lower exposure times were used (that were generated from optimal signal of a control sample) the high levels of trace dystrophin in DMD_2 are not present in the IF images and as such would not be detected during digital analysis (Fig. S6). By using a higher exposure time, the extent of trace dystrophin in this sample becomes readily apparent.

An additional novel aspect of our method is the introduction of colocalisation data and the ability to correlate levels of dystrophin in individual myofibres to a relevant protein of the DAPC. In a clinical trial setting, this allows surrogate assessment of the molecular functionality of the newly restored dystrophin following treatment. In BMD and DMD samples, there is a significant increase in the level of α-sarcoglycan and β-dystroglycan in regions of dystrophin-positive sarcolemma compared to regions of the sarcolemma that are dystrophin negative. However, the dystrophin that is present is insufficient to restore DAP levels to that of CTRL samples. This is likely because dystrophin positive sarcolemma in BMD and DMD samples contains significantly less dystrophin (as demonstrated by the significant reduction in dystrophin fluorescence) compared to dystrophin positive sarcolemma in the CTRLs.

Induction of functional dystrophin should improve sarcolemmal integrity and reduce susceptibility to myofibre necrosis and consequent regeneration in a moderately affected muscle. The ‘myosin module’ of our script enables correlative analysis between the amount of functional dystrophin and the extent of regeneration. In a majority of instances, mutations disrupting the DMD reading frame result in a severe clinical phenotype with little or no dystrophin expression in the biopsy assessed by dystrophin antibodies against a C-terminus epitope and high levels of myofibre regeneration. There are rare but well-recognised exceptions to the DMD reading frame hypothesis [32]. For instance, large in-frame deletions in the 5′ region that extend to the middle of the rod domain- such as deletions of exons 3–31, 3–25, 4–41 [34] or 4–18 [31] or even smaller deletions such as deletion of exons 3–13 are usually associated with a more severe DMD phenotype. This is despite them producing a truncated dystrophin protein with an intact C-terminus and BMD-level dystrophin in biopsies when assessed by dystrophin antibodies against a C-terminus epitope, and exemplified by the DMD2 patient in this study carrying a large in-frame deletion of exons 6–44. The clinical severity observed with these in-frame mutations may relate in part to disruption of the critical 5′ actin binding domain of dystrophin. DMD_2 presents with higher levels of dystrophin compared to DMD_1 and DMD_3 that seemingly correctly localises DAPC proteins. However, it appears to be insufficient in ameliorating the extent of regeneration as DMD_2 has greater levels of f/d-myosin positive fibres compared to DMD_1 and DMD_3. This suggests that, while a greater amount of dystrophin is present, it is at least partly non-functional despite being able to correctly localise DAPs. The extent of regeneration and the quality of restored dystrophin could serve as key quantifiable biomarkers in evaluating therapeutic response in ongoing and future DMD clinical trials.

Despite improvements on a previous version, there are certain limitations to this script. At present, the fibre recognition module is designed to recognise transverse fibres in cross-sectional orientation. Longitudinal myofibres will not be detected and as such are excluded from the analysis. To ensure the greatest percentage of fibre detection, muscle tissue biopsy blocks must be mounted in cross-sectional orientation allowing for the greatest number of transverse fibres. It is also paramount for tissue sections to be of the highest quality with as little artefactual damage as possible. While we have implemented various elements to account for certain artefactual features, heavily damaged tissue or the introduction of new artefacts will interfere with the analysis, resulting in unreliable final results. Additionally, many small newly regenerating/atrophic fibres in certain pathological samples will not be identified due to the morphometric nature of these myofibres.

## Conclusions

A novel, high throughput system for quantitative analysis of dystrophin and its associated protein complex members, alongside automated assessment of myofibre regeneration via the presence of aberrant myosin isoforms, is presented. WSI capture and automated analysis allows deep and comprehensive assessment of many thousands of myofibres within each individual muscle biopsy section using multiple parameters, such as mean fluorescence intensity, percentage positive myofibres for sarcolemmal and sarcoplasmic proteins and changes in DAPC expression in dystrophin positive and dystrophin negative regions of the sarcolemma. Combining this with our novel approach to individual myofibre background subtraction for identification of true fluorescent signal allows for accurate dystrophin quantification, even that of trace levels, and investigation of dystrophin functionality with its ability to correctly localise binding partners. Analysis of a very small cohort revealed remarkable heterogeneity in dystrophin expression patterns between different BMD and DMD patients. The ability to detect trace dystrophin, as well as quantitation of colocalised dystrophin and DAPs, will enhance our understanding of the heterogeneity of dystrophin expression in biopsies in the context of the individual genotypes in BMD and DMD, as well as a surrogate marker for molecular functionality of induced dystrophin following therapy. These aspects will be relevant to ongoing and future clinical trials in DMD.

## Supplementary information

**Additional file 1.**

## Data Availability

All data generated or analysed during this study are included in this published article.

## Abbreviations

AU: Arbitrary units
BMD: Becker muscular dystrophy
DAP: Dystrophin associated protein
DAPC: Dystrophin associated protein complex
DMD: Duchenne muscular dystrophy
F/d: Fetal and developmental
IF: Immunofluorescent
WSI: Whole slide images
