# Supplementary material for: A high–throughput digital script for multiplexed immunofluorescent analysis and quantification of sarcolemmal and sarcomeric proteins in muscular dystrophies
Source: Acta Neuropathol Commun. 2020 Apr 17;8:53. doi: 10.1186/s40478-020-00918-5 (PMC7165405; doi:10.1186/s40478-020-00918-5)
Supplement: Supplementary file 1 — Additional file 1. [file 40478_2020_918_MOESM1_ESM.docx]

**Supplementary**

**Technical Method**

The method described was developed for the analysis of whole slide images (WSI) of immunofluorescent (IF) stained transversely cut muscle sections, composed of a mask stain and two additional markers. The image analysis processing is comprised of three distinct stages: identification of muscle tissue and exclusion of artefacts; identification of muscle fibres within the tissue; and characterisation of morphological features and staining profiles of individual muscle fibres. The analysis has been implemented using Definiens Developer XD (Munich), version 2.7.0. The WSI used in this study were generated at resolution equivalent to x20 magnification at 16-bit depth

## **Identification of Tissue**

Processing of the raster image begins with downsampling to a resolution equivalent to x5 magnification. A series of greyscale images are generated from the pixel values of the original IF channels and/or applying moving average filtering (average pixel value within window of given size assigned to central pixel): *brightest* represents the highest value from the IF channels at each pixel; *brightest(101)* and *mask(11)* represent the layers *brightest* and *mask* filtered using window sizes of 101 and 11, respectively.

Initial segmentation identifies pixels above and below the mean of *brightest(101)*, to give **background** < mean ≤ **ROI**. **ROI** is grown into **background** where *mask(11)* is greater than the mean of *mask(11)* in **ROI** (threshold calculated prior to growing). This identifies all significantly stained regions. Any **background** enclosed by **ROI** is removed into **ROI**; the **ROI** object(s) now represent whole tissue areas (FigS1(a)). **ROI** objects are then analysed to identify non-tissue related staining; **ROI** with low standard deviation in *mask(11)* that contain 5 or less elliptically shaped objects delineated by mask staining are removed into **background**. Regions of **ROI** with dense mask staining are classified as **artefact** and excluded from proceeding stages.

## **Identification of Muscle Fibres**

Muscle fibre identification is performed on the **ROI** objects at the highest available resolution – the use of the native resolution allows maximum identification of small fibres. A fine (7) and coarse (251) filter are applied to the *mask* layer, giving *mask(fine)*, a minimally modified representation of *mask*, and *mask(coarse)*, which represents the background staining character, respectively. These are combined to give *mask(modified-251)* which represents *mask(fine)* - *mask(coarse)* where the value of this calculation is greater than 0 (all negative pixels recorded as 0); a single threshold of 0 is then used to identify significant *mask* staining within the **ROI**, giving **non-mask** < 0 ≤ **mask** (FigS1(b)).

Once the initial sarcolemma identification has been performed further processing, such as threshold and morphology based segmentation, is targeted by identifying those sarcoplasm regions that fall within the morphological tolerances of muscle fibres, i.e. circularity/elliptic fit, width/length, concavity.

It is important to strike a balance between identification of as many muscle fibres as possible and avoiding the false identification of non-fibre objects. However it is possible to identify additional fibres not fully described by the modified images. Additional processing performed to improve the fibre identification include: shrink and grow – shrinking of **non-mask** by 10 pixels to a **temporary** object class, then growing **non-mask** to fill **temporary**. Where there is a small gap in the mask staining, **non-mask** objects are cleaved in two, introducing a separating boundary between **non-mask** objects (Fig2S); native stain thresholding: **mask** stain is extended/grown into **non-mask** where *mask* is greater than the given threshold (70^th^ centile of *mask* in the **ROI**).

Once all available **sarcoplasm** objects have been identified the sarcolemma element of a fibre is added by coating each **sarcoplasm** with an enclosing layer of **sarcolemma** into **mask**. The depth of **sarcolemma** growth is limited by three factors: reaching the border of **mask**; abutting against another **sarcolemma** object from a neighbouring fibre (all **sarcolemma** objects are grown by one pixel layer at a time so the available **mask** is split evenly); showing a decrease in *mask* intensity after reaching a thickness of 3µm; maximum of 6 additional layers of pixels giving a maximum thickness of 6µm. Each **sarcoplasm** and its associated **sarcolemma** are combined to give a **fibre**, which is formed of and describes both constituents (Fig3S).

## **Morphological and Immunohistological Characterisation of Muscle Fibres**

For each **fibre** a series of morphological descriptors and staining characteristics are calculated and recorded. The morphological descriptors are fibre area, sarcoplasm area, sarcolemma area, fibre width, sarcoplasm width, and mean and max sarcolemma thickness. The staining characteristics include both direct measurements of staining intensity within the sarcolemma and sarcoplasm for all image layers, and empirically enhanced characteristics. The reporting of stain properties is adapted to the two stain combinations under investigation: mask with primary and secondary sarcolemma stain; and mask with a primary sarcolemma and secondary sarcoplasmic stain.

## **Sarcolemmal Stains**

For the sarcolemma stain(s) a background subtraction method is used to identify only significant levels of staining as positive. This identifies an exclusion threshold for each fibre individually, based on the intensity of staining within the sarcoplasm, as it is observed that the sarcoplasm always shows a low level of marker expression compared with non-fibre regions, and this low level of marker expression is always equal to or lower than the level within the sarcolemma.

The threshold (*marker*+ve) used to identify positive staining within the **sarcolemma** of each fibre is calculated from the **sarcoplasm** region, based on the stain intensity of the marker under investigation: $\bar{marker}\left[ \boldsymbol{sarcoplasm} \right]+(5\times\delta(marker)\left[ \boldsymbol{sarcoplasm} \right])$. A minimum threshold (min(*marker*+ve)) is then calculated as the 2^nd^ centile of *marker*+ve for all fibres – this is the value of *marker*+ve that identifies the fibres with the lowest 2% of threshold values.

For each fibre the **sarcolemma** is segmented as **negative** < *marker*+ve ≤ **positive** (where *marker*+ve is less than min(*marker*+ve), min(*marker*+ve) is used; this avoids the false identification of positivity in fibres with very low levels of staining). Two positive coverage measurements are then calculated:

- Area Positivity – the area of **positive** as a proportion of the area of **sarcolemma**;
- Circumference Positivity – the sum of (border length-2)/2 for each **positive** object in a fibre, as a proportion of the circumference of **sarcolemma**.

The circumference positivity is calculated using pixels. I.e. a single pixel will have a border of 4, 2 pixels a border of 6, a 4 pixel square a border of 8, etc. It is not necessary to convert to a distance unit as the subtraction of 2 is performed to remove the effect of the end pixel, i.e. (4-2)/2 =1 for the single pixel, (6-2)/2 = 2, (8-2)/2 – 3 for the larger square. Whilst Area Positivity provides a metric that can be compared with most other methods of measurement, it was found to provide artificially low positivity scores due to the relatively high thickness of sarcolemma as delineated by mask staining, compared with the relatively narrow banding of marker expression that a pathologist would recognise as significant. This means that a fibre with a thin band of staining around 80% of the sarcolemma could be recorded as having the same level of positivity as a fibre with a thick band of staining around 25% of the sarcolemma. The Circumference Positivity has been shown to provide both quantitatively and qualitatively robust measurement of positive coverage, providing a high degree of consistency between similarly scored fibres and with pathologist classification.

The intensity of the primary and, where the secondary stain is for a sarcolemma located marker, the secondary marker, are calculated in the **positive** and **negative** objects contained within the **sarcolemma** for each fibre (Fig S4). This provides a direct measurement of the sarcolemma staining within the sarcolemma region classified as positive and negative for the primary sarcolemma stain, which is a direct measurement of colocalisation.

Each **fibre** is then classified into one of 4 classes according to the Circumference Positivity: **0-25%**, **25-50%**, **50%-75%** and **75-100%** coverage, representing negative, low positivity, medium positivity and high positivity, respectively.

## **Sarcoplasmic Stains**

The classification of sarcoplasmic staining is binary rather than graduated, with expression localised to the sarcoplasmic compartment rather than present throughout the myofibre structure. Therefore the identification of a suitable threshold to identify positive fibres is largely an attempt to account for non-specific staining and auto-fluorescence.

To identify a suitable fixed threshold, the fibre counts were generated for bins of 1 (8-bit depth) according to the average stain intensity within the sarcoplasm for the manually classified fibres. The resultant histogram described two distinct distributions for positive and negative fibres with a small proportion of crossover. The mid-point of this crossover region (33 (8-bit depth)) was identified as a suitable threshold and subtracted from each pixel. Fibres are then classified based on the average adjusted stain intensity within sarcoplasm, giving **negative** ≤ 0 < **positive**.


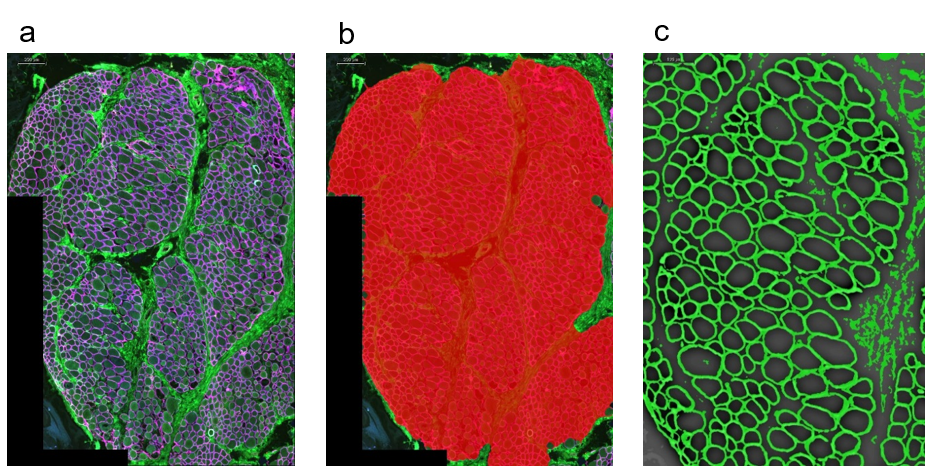


***Fig.S1***

*Identification of tissue, shown in red* ***(B)****, and sarcolemmal mask staining, shown in green* ***(C)****, within the WSI and marked area of* ***(A)*** *respectively*


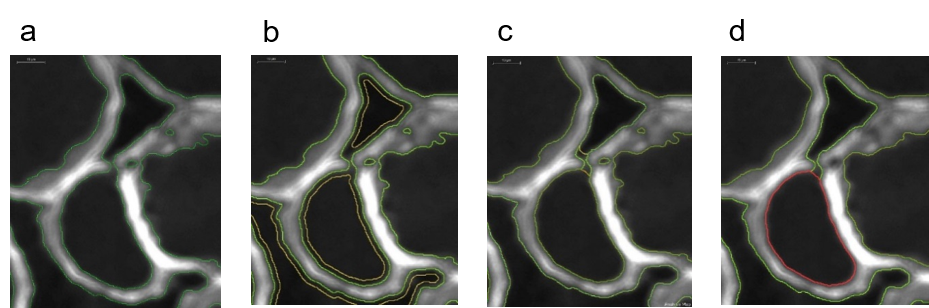


***Fig.S2***

*An example of the shrink and grow method used to identify muscle fibres that are not fully outlined by the mask stain. The non-fibre object* ***(A)*** *is shrunk* ***(B)*** *giving two separated objects. These are then grown* ***(C)****, any objects that identify as muscle fibres are then reclassified (red outline) and remaining objects merged* ***(D)***


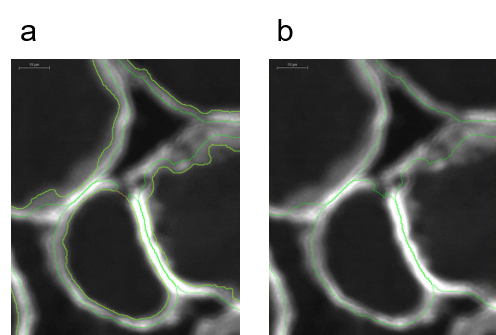


***Fig.S3***

*Sarcolemma (green) and sarcoplasm (yellow)* ***(A)*** *are combined to give each muscle fibre* ***(B)***


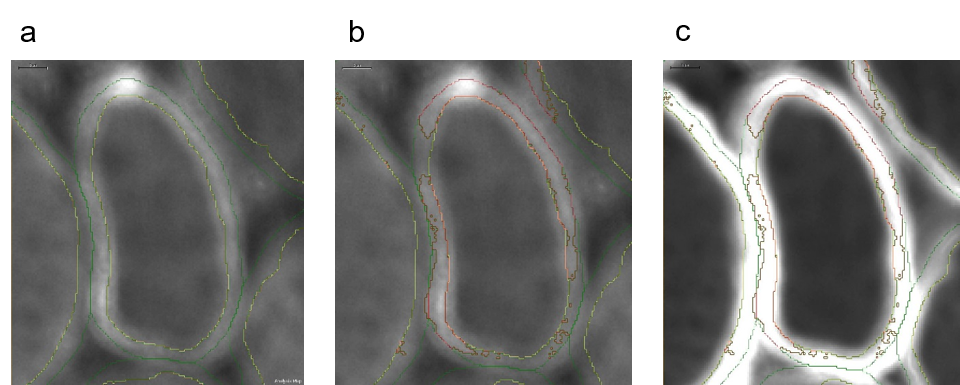


***Fig.S4***

*For the primary sarcolemmal marker, the sarcolemma (green) of each myofibre* ***(A)*** *is segmented based on thresholds calculated from the individual fibres sarcoplasm (yellow) stain profile, to give positive and negative sarcoplasm (****B****– red and green respectively). The intensity of the secondary sarcolemmal marker is then measured in these primary marker positive and negative regions to measure colocalisation* ***(C)***


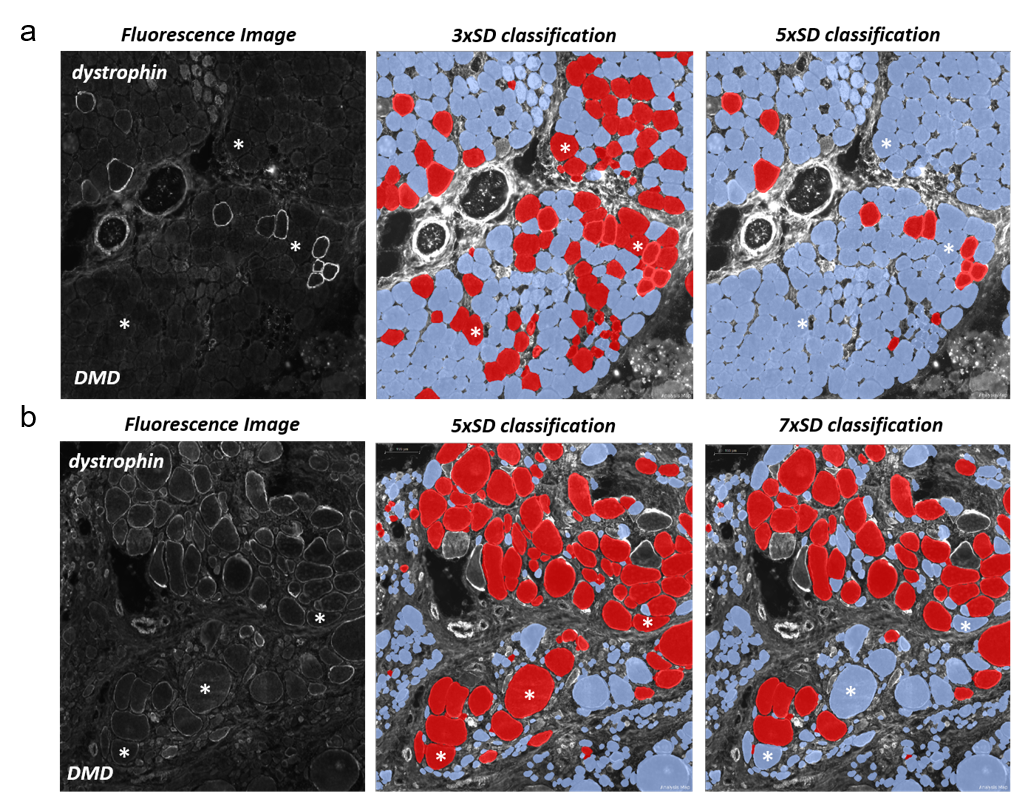


***Fig.S5***

***(A)*** *Dystrophin classification from an example region of a DMD sample at 3x and 5x standard deviation using the dynamic threshold. Positive fibres are highlighted in red and negative fibres highlighted in blue. Fibres marked with * are example fibres that have been incorrectly classified positive at 3x SD and correctly classified as negative at 5x SD as confirmed by a pathologist.* ***(B)*** *Dystrophin classification from an example region of a DMD sample at 5x and 7x standard deviation using the dynamic threshold. Positive fibres are highlighted in red and negative fibres highlighted in blue. Fibres marked with * are example fibres that have been incorrectly classified negative at 7x SD and correctly classified as positive at 5x SD as confirmed by a pathologist*


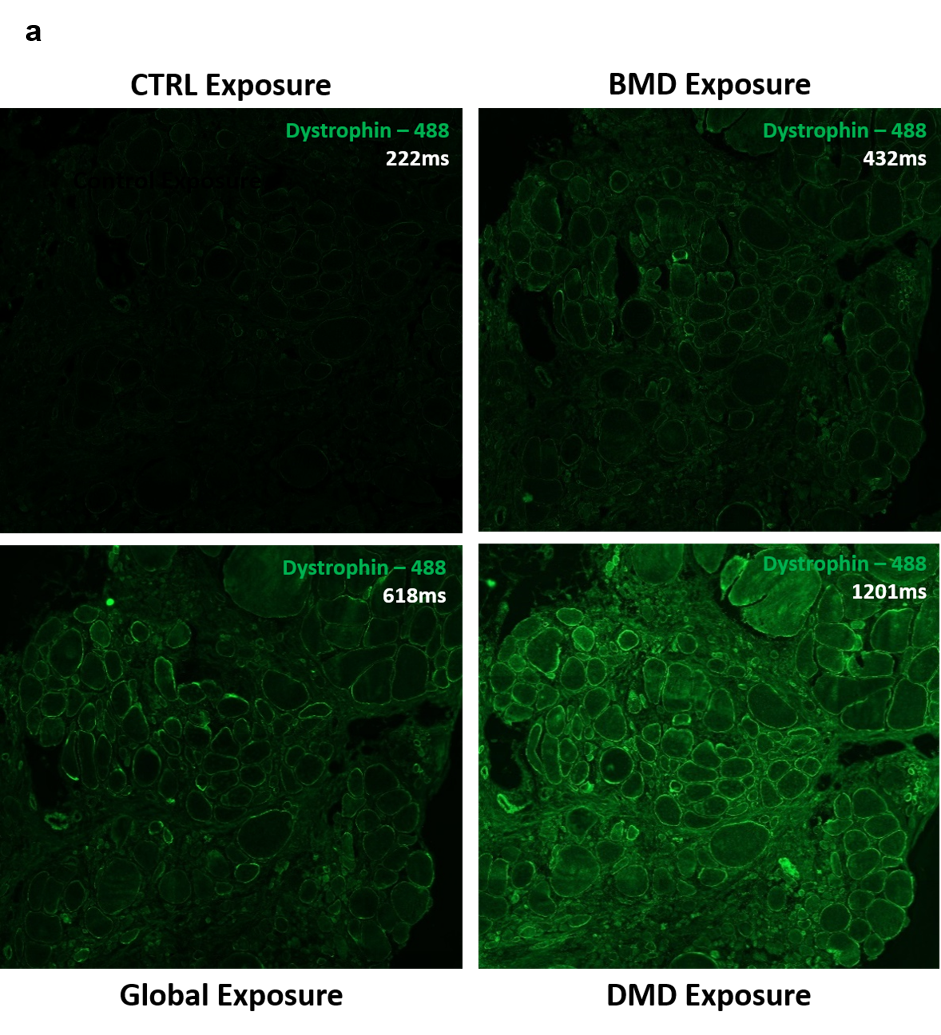


***Fig.S6***

*Dystrophin immunostaining of DMD_2 at 4 different exposure times (222ms, 432ms, 618ms and 1201ms) calculated from average auto exposure values of CTRL, BMD, DMD and all samples together*
